# Editorial: Legume root diseases

**DOI:** 10.3389/fpls.2024.1393326

**Published:** 2024-03-20

**Authors:** Marie-Laure Pilet-Nayel, Clarice J. Coyne, Christophe Le May, Sabine Banniza

**Affiliations:** ^1^ IGEPP, INRAE, Institut Agro, Univ Rennes, Le Rheu, France; ^2^ USDA-ARS Plant Germplasm Introduction & Testing Research, Pullman, WA, United States; ^3^ IGEPP, INRAE, Institut Agro, Univ Rennes, Rennes, France; ^4^ Crop Development Centre/Department of Plant Sciences, University of Saskatchewan, Saskatoon, SK, Canada

**Keywords:** soil-borne pathogens, root rot complex, disease resistance and breeding, plant-pathogen-microbe interaction, integrated disease management

Legume crop development is a major challenge worldwide for sustainable agriculture and food security. In particular, legume root diseases are economically important, affecting large areas of crop production in many countries worldwide. Root rots, caused by *Aphanomyces euteiches, Rhizoctonia solani, Fusarium* species, and wilts, caused by several formae speciales of *Fusarium oxysporum*, are some of the most destructive soil-borne diseases of cultivated legumes including pea, chickpea, lentil, soybean, bean, faba bean, lupin, and alfalfa. A number of control strategies have been developed including resistance breeding, cultural practices and chemical control. However, root diseases management remains challenging, especially due to difficult accessibility to the soil horizon and implementation of most control methods has been hard to achieve or resulted in incomplete protection. Collaborative and multidisciplinary research is needed to develop effective integrated control strategies against these diseases.

International Workshop meetings were initiated on legume root diseases, with the first held in 2002 in Rennes, France. As the community interest in this topic has grown, the meetings have performed an important role in the international promotion and discussion of results on the research and scientific achievements in this field. They have also been important in supporting the scientific community and helping breeders and stakeholders with funding applications and partnerships. The eighth International Legume Root Diseases (ILRD8) workshop was held online on August 23-26, 2022 (https://workshop.inrae.fr/ilrd8/) and gathered nearly 100 scientists from different continents of the world. The workshop provided the opportunity to develop this Frontiers Research Topic bringing together a collection of the latest quality articles that report on recent advances in research on legume root diseases. Manuscripts of this Research Topic address the various areas of legume root diseases research, including disease survey, pathogen identification and diversity (four articles), disease resistance and breeding (four articles), plant-pathogen-microbe interactions (three articles) and integrated disease management (five articles).

Root disease survey, pathogen identification and diversity were reported on faba bean, chickpea and pea. In China, Long et al.  first reported black root rot on faba bean caused by *Berkeleyomyces rouxiae*. These authors confirmed and expanded the broad host range of *B. rouxiae* in legumes and identified moderately resistant faba bean cultivars. In a survey undertaken across Germany from 2016-2019, Šišić et al.  identified significantly higher frequencies of *Fusarium redolens* and *Didymella pinodella* in roots of faba bean from organic fields compared with conventional fields, and lower frequencies of *F. avenaceum*, *F. tricinctum* and *F. culmorum.* These differences in frequency of isolation of several species were linked to the presence of legumes in organic field rotations compared to their absence in conventional fields. In a two year-survey in Saskatchewan, Canada, Armstrong-Cho et al. identified prevalence of *Fusarium* species, including *F. redolens*, *F. solani* and *F. avenaceum* obtained by root isolations and molecular tests, associated with root rot of chickpea. Isolates of *F. avenaceum* were the most aggressive of the *Fusarium* isolates identified on chickpea. In France and in the United States, Moussart et al. characterized pathotypes of pea-infecting isolates of *Aphanomyces euteiches* recovered from pea breeding nurseries. The authors identified a reduced virulence diversity and a higher vs. lower aggressiveness on pea vs. *Medicago truncatula* of French isolates compared to American isolates, suggesting the role of legume crop history as a driver of pathogen population evolution.

Studies on disease resistance and breeding for root diseases were addressed on chickpea, pea and *Medicago* spp. Bithell et al. showed that *Phytophthora medicaginis* soil DNA concentrations, as a parameter for quantifying pathogen proliferation, was correlated to disease severity and yield loss and thus could be used to screen for partial resistance in chickpea. In pea, Kälin et al. analyzed the level of partial resistance to *Aphanomyces euteiches* from the landrace PI 180693 in back-crossed pea breeding lines. They identified variability in pea responses to *Aphanomyces euteiches* isolates in controlled condition tests, that depended on their virulence levels. They reported that partial resistance could to be less effective towards root rot caused by *Phytophthora pisi* in field assays. Leprévost et al. provided a meta-analysis of the diversity of Quantitative Trait Loci (QTL) for resistance to *Aphanomyces euteiches* in different sources of resistance in pea, by integrating a novel QTL mapping study in two advanced backcross populations with previous QTL analyses and genome-wide association study (GWAS). The authors identified ten consistent genetic regions and a diversity of resistance haplotypes in new sources of resistance which will be helpful to support breeding efforts for resistant pea varieties. In a *Medicago truncatula* collection, Fartash et al. identified by GWAS new genetic loci associated with resistance towards an Iranian strain of *Verticillium alfalfae* adapted to higher temperature (25°C), compared to loci identified previously with another strain adapted to lower temperature (20°C). The authors suggested that a simple shift in temperature combined with a new pathogen strain could change the architecture of genetic control of resistance to the pathogen.

Research advances on plant-pathogen-microbe interactions were also reported in this Research Topic with a special application to legume-*Aphanomyces euteiches* interactions. Fortier et al. reviewed the role of the root border cells in plant root defense against soilborne pathogens, with a focus on root rot disease caused by *Aphanomyces euteiches*. The root extracellular trap, a thick mucilage layer at the interface between the soil and the root, was reported to be enriched in antimicrobial compounds including defense-related proteins, secondary metabolites and glycan-containing molecules. *In vitro* assays showed that arabinogalactan proteins isolated from pea root cap and border cells were able to attract zoospores of *Aphanomyces euteiches* and inhibit subsequent cyst germination. Kiselev et al. identified 35 active extracellular microbial proteases using Activity Based Protein Profiling and mass spectrometry (ABPP-MS) on apoplastic fluids isolated from pea roots infected by *Aphanomyces euteiches*. These novel active modular extracellular eukaryotic proteases are relevant targets as potential pathogenicity factors in the *Aphanomyces* genus. Hashemi et al. reported that *Pythium oligandrum*, a soilborne oomycete used as a biological control agent, promoted plant growth in pea and *Medicago truncatula* and protected them against infection by *Aphanomyces euteiches*. *P. oligandrum* also activated plant immunity in *M. truncatula* roots, notably upregulating genes involved in the biosynthesis of antimicrobial compounds and enhancing the production of phytoalexins, medicarpin and formononetin, but it did not impair symbiotic interactions.

Integrated root disease management was reported in several studies on pea, lentil, faba bean, bean and lucerne. For prophylactic management, Chatterton et al. developed molecular methods for quantifying soil inoculum to assess the risk of pea root rot in field soil samples. A significant linear relationship was determined between DNA concentrations of *Aphanomyces euteiches* measured in soil by digital droplet PCR and quantitative PCR and oospore inoculum concentration, although it was dependent on soil types and other pathogens. For biological management, Hubbard et al. examined the impacts of nitrogen fertilization and a commercial arbuscular mycorrhizal fungal (AMF) inoculant on pea and lentil plant health and production in three fields in Canada. The authors identified no impact of the AMF on pea and lentil *Aphanomyces* and *Fusarium* root rot severity. Nitrogen fertilization showed variable effects on root rot for pea and lentil crops, depending on field trial sites. Pisarčik et al. reported that application of the mycoparasitic oomycete *Pythium oligandrum* as a biocontrol agent, in both the autumn and intensive schedules in the field, showed no effect on lucerne root disease score when attacked by *Fusarium* and *Verticillium*. All these authors suggested that both environmental and soil characteristics can affect biological treatment efficacy substantially. For integrated disease management, Yu et al. reviewed disease management methods for root rots on faba bean in China, caused by multiple pathogens among which *Fusarium* spp. were the most prevalent. These methods include intercropping with non-host crops, the most widely utilized control method, applying rational nitrogen, and treating seeds with chemical or bio-seed treatments. Naseri and Kakhki  evaluated and modeled the effect of different cultivars, planting dates and weed control methods on progression of common bean growth, *Rhizoctonia* root rot and weed development in a two-year trial in Iran. The authors showed that late planting restricted *Rhizoctonia* root rot progress, thus improving bean yield, and identified model parameters for monitoring bean-disease-weed development and predicting bean productivity.

All of these research results clearly illustrate the need and opportunities to both deepen knowledge in targeted disciplines and integrate them into a more comprehensive, multidisciplinary system, so that they translate into impactful plant protection innovations in the field. Further communications within the scientific community through international legume root disease workshops, such as the ILRD9 hosted on September 18, 2023 in Granada, Spain, and the upcoming ILRD10, will be very useful in accelerating progress towards the development of integrated, effective, sustainable and environment-friendly management methods for legume root diseases worldwide.

## Author contributions

M-LP-N: Writing – original draft. CC: Writing – review & editing. CM: Writing – review & editing. SB: Writing – review & editing.

